# High Dietary Sodium Intake Assessed by Estimated 24-h Urinary Sodium Excretion Is Associated with NAFLD and Hepatic Fibrosis

**DOI:** 10.1371/journal.pone.0143222

**Published:** 2015-11-16

**Authors:** Ji Hye Huh, Kyong Joo Lee, Jung Soo Lim, Mi Young Lee, Hong Jun Park, Moon Young Kim, Jae Woo Kim, Choon Hee Chung, Jang Yel Shin, Hyun-Soo Kim, Sang Ok Kwon, Soon Koo Baik

**Affiliations:** 1 Division of Endocrinology and Metabolism, Department of Internal Medicine, Yonsei University Wonju College of Medicine, Wonju City, Korea; 2 Division of Gastroenterology and Hepatology, Department of Internal Medicine, Yonsei University Wonju College of Medicine, Wonju City, Korea; RWTH Aachen, GERMANY

## Abstract

**Background:**

Although high sodium intake is associated with obesity and hypertension, few studies have investigated the relationship between sodium intake and non-alcoholic fatty liver disease (NAFLD). We evaluated the association between sodium intake assessed by estimated 24-h urinary sodium excretion and NAFLD in healthy Koreans.

**Methods:**

We analyzed data from 27,433 participants in the Korea National Health and Nutrition Examination Surveys (2008–2010). The total amount of sodium excretion in 24-h urine was estimated using Tanaka’s equations from spot urine specimens. Subjects were defined as having NAFLD when they had high scores in previously validated NAFLD prediction models such as the hepatic steatosis index (HSI) and fatty liver index (FLI). BARD scores and FIB-4 were used to define advanced fibrosis in subjects with NAFLD.

**Results:**

The participants were classified into three groups according to estimated 24-h urinary excretion tertiles. The prevalence of NAFLD as assessed by both FLI and HSI was significantly higher in the highest estimated 24-h urinary sodium excretion tertile group. Even after adjustment for confounding factors including body fat and hypertension, the association between higher estimated 24-h urinary sodium excretion and NAFLD remained significant (Odds ratios (OR) 1.39, 95% confidence interval (CI) 1.26–1.55, in HSI; OR 1.75, CI 1.39–2.20, in FLI, both *P* < 0.001). Further, subjects with hepatic fibrosis as assessed by BARD score and FIB-4 in NAFLD patients had higher estimated 24-h urinary sodium values.

**Conclusions:**

High sodium intake was independently associated with an increased risk of NAFLD and advanced liver fibrosis.

## Introduction

Non-alcoholic fatty liver disease (NAFLD) is the most common metabolic liver disease with a prevalence as high as 30% in developed countries, and its incidence has been increasing rapidly along with the rise in obesity [1]. NAFLD is generally recognized as a major risk factor for various metabolic disorders including type 2 diabetes mellitus, dyslipidemia and cardiovascular disease [2, 3]. Furthermore, NAFLD is clinically important because a relevant proportion of patients, especially those with nonalcoholic steatohepatitis (NASH), may develop cirrhosis and its complications [4]. Considering the close relationship between NAFLD and serious metabolic disorders, it is important to identify specific modifiable risk factors for NAFLD and progression to NASH in clinical practice.

High consumption of dietary salt is suggested to be related to various metabolic disorders, including hypertension and cardiovascular disease [5]. Increased dietary sodium intake has also been reported to be associated with insulin resistance and type 2 diabetes mellitus [6]. Furthermore, recent studies report that high salt intake, reflected by higher 24-h urinary sodium excretion values, is associated with metabolic syndrome, obesity and sarcopenia [7–9]. Although growing evidence indicates that high sodium intake deleteriously influences body phenotype and metabolic disease, few studies have explored an association between dietary sodium intake and NAFLD.

Measurement of sodium excretion from 24-h urine collection has become the preferred method for assessing individuals’ dietary salt intake in population surveys because dietary recall is difficult and inaccurate. However, 24-h urine collection procedures are expensive and inconvenient for study participants. For this reason, estimating sodium excretion by spot urine specimens has been widely used to assess individual sodium intake [10]. Recent studies have shown that sodium excretion values estimated this way correlate well with exact sodium excretion values measured via 24-h urine collection, as well as with actual individual salt intake [11, 12]. Tanaka’s equation was developed to estimate 24-h urinary sodium excretion from spot urine specimens collected at any time, using Japanese data from the INTERSALT study [13], which demonstrated that sodium excretion estimated using Tanaka’s equation was significantly correlated with measured sodium excretion using 24-h urine collection [13]. From this background, the aim of our study was to determine whether high salt intake assessed by estimated 24-h urinary sodium excretion is associated with NAFLD in a healthy Korean population. We further investigated whether high sodium intake is also associated with advanced liver fibrosis in subjects with NAFLD.

## Materials and Methods

### Study population and design

Participants in the 2008–2010 Korea National Health and Nutrition Examination Surveys (KNHANES) were recruited for this study. The KNHANES has been periodically performed by the Division of Chronic Disease Surveillance of the Korean Centers for Disease Control and Prevention since 1998. The purpose of the KNHANES was to assess the health and nutritional status of the civilian, non-institutionalized population of the Republic of Korea. The KNHANES was a cross-sectional and nationally representative survey comprised of a health interview survey, a nutrition survey, and a health examination survey [14]. Data were collected by household interviews and by direct, standardized physical examinations conducted in mobile examination centers. Nutritional status including dietary information and medical history were evaluated using a 24-h recall method. Regular exercise was indicated as “yes” when the subject exercised for more than 20 min at a time and more than three times per week. We excluded subjects who met the following criteria: alcohol consumption >140 g/week for men and 70 g/week for women (N = 1,928); positive serologic markers for hepatitis B (N = 167) or hepatitis C virus (N = 31); or the presence of liver cirrhosis (N = 67). A diagnosis of cirrhosis was established by histologic criteria or clinical and ultrasonographic findings. Of all survey participants who met the inclusion criteria, 27,433 participants aged 25 years or older were recruited for the present study. All participants were provided with written informed consent to participate in this survey, and we received the data in anonymized form. The study was carried out in accordance with the ethical standards of the Helsinki Declaration.

### Measurements

Body fat was measured with a dual-energy X-ray absorptiometer (QDR 4500A; Hologic Inc., Waltham, MA, USA). Well-trained observers manually measured blood pressure with a mercury sphygmomanometer (Baumanometer; Baum, Copiague, NY, USA). During the survey, a random urine sample was collected. All samples were refrigerated and transported to the central laboratory within 24 h. Urinary sodium levels were measured using the ion-selective electrode method. Serum and urine creatinine levels were assessed with the Jaffe reaction and measured with an automatic analyzer (ADVIA 1650 system; Bayer Health Care, Tarrytown, NY, USA). Blood samples were immediately refrigerated, transported to the Central Testing Institute in Seoul, Korea, and analyzed within 24 h. The serum levels of creatinine and the lipid and liver enzyme profiles were determined using a Hitachi 7600 automated chemistry analyzer (Hitachi, Tokyo, Japan) using the indicated methods. Fasting insulin (INS-IRMA; Biosource, Nivelles, Belgium) was measured by an immunoradiometric assay. Homeostasis model assessment of insulin resistance (HOMA-IR) values were calculated using the following formula: fasting [plasma glucose (mg/dL) × fasting insulin (mIU/mL)]/22.5 [15].

### Estimation of 24-h urinary sodium excretion values

The 24-h urinary sodium values were estimated from the sodium and creatinine values of random urine samples using Tanaka’s equation [13], as follows: Estimated 24-h urinary Na excretion (mmol/day) = 21.98×U_Na_/U_Cr_×{-2.04×age+14.89×weight (kg)+16.14×height (cm) -2244.45}^0.392^. Participants were classified into three groups according to the estimated 24-h urinary Na excretion tertiles.

### Definitions of hepatic steatosis and advanced fibrosis

NAFLD was defined using two previously validated fatty liver prediction models: (1) Hepatic steatosis index (HSI) = 8 × (ALT/AST ratio) + BMI (+2, if female; +2, if diabetes mellitus)[16] and (2) fatty liver index (FLI) = [e^0.953^×log_e_ (TG) + 0.139×BMI+0.718×log_e_ (GGT) +0.053×waist circumference–15.745] / [1+e^0.953^× log_e_ (TG) + 0.139×BMI+0.718×log_e_ (GGT) + 0.053×waist circumference–15.745] × 100, with triglycerides measured in mmol/l, GGT in U/l and waist circumference in cm [17]. We defined NAFLD as HSI of 35 or higher and/or FLI of 60 or higher. The BARD score [18] and FIB-4 index [19] were selected as a surrogate indices for defining severity of NAFLD (hepatic fibrosis). BARD and FIB-4 were calculated only in subjects with NAFLD defined using FLI (FLI ≥60).

### Statistical analyses

Statistical analyses were conducted using PASW Statistics, version 20 (SPSS Inc., Chicago, IL, USA). One-way analysis of variance (ANOVA) followed by Scheffé post hoc comparison was used to compare differences in clinical characteristics between groups. For categorical variables, a Chi square test was used to compare frequencies between groups. Multiple logistic regression analysis was used to examine the adjusted odds ratios of estimated 24-h urinary sodium excretion tertiles for the presence of NAFLD. A P-value less than 0.05 was considered statistically significant.

## Results

### Patient characteristics

The demographic and clinical characteristics of the patients, who were classified into three groups according to estimated 24-h urinary sodium excretion tertiles, are shown in Table 1. The mean subject age was 51.52 ± 15.71 years (range, 25–98 years). The prevalence of hepatic steatosis as assessed by FLI was 39.8% and the prevalence of hepatic steatosis as assessed by HSI was 64.3%. The prevalence of hepatic steatosis was significantly higher in the highest estimated 24-h urinary sodium excretion tertile compared with the other tertile groups. As the estimated 24-h urinary sodium excretion increased, subjects were more likely to be male, hypertensive, insulin resistant and diabetic. Obesity indices, including body weight, BMI, waist circumference and percentage body fat also showed a gradual increase as the estimated 24-h urinary sodium excretion increased. Fasting glucose, total cholesterol, LDL cholesterol, HDL cholesterol, TG and AST were significantly higher in the highest estimated 24-h urinary sodium excretion group compared with those in the lowest estimated 24-h urinary sodium excretion group. The highest estimated 24-h urinary sodium excretion group also had higher levels of ALT than the other groups (T1: 20.84±18.23, T2: 21.03±14.79, T3: 22.26±15.93, *P* < 0.001).

**Table 1:** Characteristics of the study population according to tertiles of estimated 24-h sodium excretion.

	Estimated 24-h sodium excretion (mEq/day)			
	T1 (35.97–127.94)	T2 (127.95–158.25)	T3 (158.26–450.92)	*P*-value
N	9144	9145	9144	
E24UNA (mEq/day)	106.70±16.47§ †	142.84±8.65§ ‡	188.49±22.64† ‡	<0.001
Age (year)	49.31±15.74§ †	51.65±14.92§ ‡	54.85±14.63† ‡	<0.001
Sex, male (%)	3822 (41.8%)†	3915 (42.8%)	4035 (44.1%)†	0.006
Weight (Kg)	60.14±11.25§ †	62.05±11.03§ ‡	64.76±11.25† ‡	<0.001
BMI (kg/m^2^)	23.07±3.25§ †	23.64±3.13§ ‡	24.52±3.34† ‡	<0.001
Waist circumference (cm)	84.07±9.19†	84.9±8.38‡	86.31±8.48† ‡	<0.001
Body fat (%)	28.39±7.67	28.80±7.53	29.35±7.80	<0.001
Current smoking (%)	476 (40.8%)	381(32.4%)	369(31.4%)	<0.001
Regular exercise (%)	218 (18.7%)	212 (118%)	215 (18.3%)	0.919
Education				<0.001
Elementary	2166 (24%)	2356 (26.2%)	3068 (34%)	
Middle school	876 (9.7%)	1034 (11.5%)	1225 (13.6%)	
High school	2810 (31.1%)	2874 (31.9%)	2669 (29.5%)	
College	3172 (35.2%)	2750 (30.5%)	2073 (22.9%)	
Daily total energy intake (kcal)	1899.1±846.4§ †	1953.4±824.2§	1931.3±814.0†	<0.001
Daily fat intake (g)	37.59±31.12†	37.25±29.27‡	34.78±29.44† ‡	<0.001
Daily carbohydrate intake (g)	311.53±122.48§ †	324.59±124.50§	327.06±125.44†	<0.001
SBP (mmHg)	116.63±16.27§ †	120.37±17.22§ ‡	125.22±18.48† ‡	<0.001
DBP (mmHg)	75.15±10.18§ †	76.85±10.51§ ‡	78.55±10.67† ‡	<0.001
Fasting glucose (mg/dL)	97.21±24.41†	97.36±19.93‡	99.92±23.37† ‡	<0.001
HOMA-IR	2.12±0.96†	2.31±1.68	2.4±1.17†	0.047
Total cholesterol (mg/dL)	190.16±36.17†	190.33±35.87†	192.11±36.72† ‡	<0.001
LDL cholesterol (mg/dL)	114.73±35.52	115.2±31.41	116.84±31.65	0.04
HDL cholesterol (mg/dL)	53.20±12.83§ †	52.18±12.67§ ‡	51.34±12.45† ‡	<0.001
Triglyceride (mg/dL)	123.03±86.33§ †	132.02±96.41§ ‡	145.55±112.04† ‡	<0.001
AST (IU/L)	22.02±11.52†	22.01±10.23‡	22.98±10.79† ‡	<0.001
ALT (IU/L)	20.84±18.23†	21.03±14.79‡	22.26±15.93† ‡	<0.001
GGT(mg/dL)	31.17±40.31	30.26±32.09‡	32.42±40.74‡	0.018
HTN (%)	2653 (29.4%)§ †	2928 (32.4%)§ ‡	3720 (41.2%)† ‡	<0.001
HTN medication (%)	1870 (20.6%)	1744 (19.2%)‡	2153 (23.8%) ‡	<0.001
DM (%)	566 (6.3%)†	631(7.0%)‡	946 (10.5%)† ‡	<0.001
Hepatic steatosis				
Assessed by FLI	514 (10.1%)†	587 (11.8%)‡	860 (17.9%)† ‡	<0.001
Assessed by HSI	1532 (17.3%)§ †	1793 (20.3%)§ ‡	2348 (26.7%)† ‡	<0.001

Data presented as mean ± standard deviation or n (%) for categorical variables

^§^: The difference between 1^st^ and 2^nd^: p <0.05 after ANOVA followed by Scheffé post hoc comparison

^†^: The difference between 1^st^ and 3^rd^: p <0.05 after ANOVA followed by Scheffé post hoc comparison

^‡^ The difference between 2^nd^ and 3^rd^: p <0.05 after ANOVA followed by Scheffé post hoc comparison

E24UNA, Estimated 24-hour urine sodium excretion; BMI, body mass index; ASM, appendicular skeletal mass; SBP, systolic blood pressure; DBP, diastolic blood pressure; LDL, low-density lipoprotein; HDL, high-density lipoprotein; AST, aspartate aminotransaminase; ALT, alanine aminotransferase; GGT, gamma-glutamyl transferase; HTN, hypertension; FLI, fatty liver; HSI, hepatic steatosis index

### Prevalence of NAFLD according to estimated 24-h urinary sodium excretion

After adjustment for all confounding factors, including age, sex, percentage of body fat, smoking status, regular exercise, educational level, diabetes, daily total energy intake, anti-hypertension medication, HOMA-IR, triglycerides and serum creatinine, the highest estimated 24-h urinary sodium excretion tertile group had a significantly higher risk for the presence of NAFLD than other groups regardless of NAFLD prediction model (HSI model; Odds ratio (OR) 1.39, 95% confidence interval (CI) 1.26–1.55, *P* < 0.001, FLI model; OR 1.75, CI 1.39–2.20, *P* < 0.001, Table 2). Furthermore, each standard deviation increase in estimated 24-h urinary sodium excretion was associated with a 21~29% increase in the risk of NAFLD (*P* < 0.001).

**Table 2:** Adjusted odds ratios (ORs) with 95% confidence interval (CI) of non-alcoholic fatty liver disease (NAFLD) assessed by different predictive models according to the tertiles of estimated 24-h sodium excretion and per standard deviation (SD) of estimated 24-h sodium excretion.

	NAFLD assessed by HSI		NAFLD assessed by FLI	
	ORs (95% CI)	*P*	ORs (95% CI)	*P*
Estimated 24-h sodium excretion (mEq/day)				
T1	reference		reference	
T2	1.14(1.03–1.27)	0.014	1.14 (0.90–1.45)	0.281
T3	1.39 (1.26–1.55)	<0.001	1.75 (1.39–2.20)	<0.001
Per SD	1.21(1.16–1.26)	<0.001	1.29 (1.19–1.41)	<0.001

Adjusted for age, sex, percentage of body fat, smoking status, regular exercise, educational level, diabetes, daily total energy intake, daily fat intake, daily carbohydrate intake, anti-hypertension medication, log transformed HOMA-IR, log transformed triglycerides and serum creatinine

### Association between estimated 24-h urinary sodium excretion and hepatic fibrosis in subjects with NAFLD

**Fig 1:**
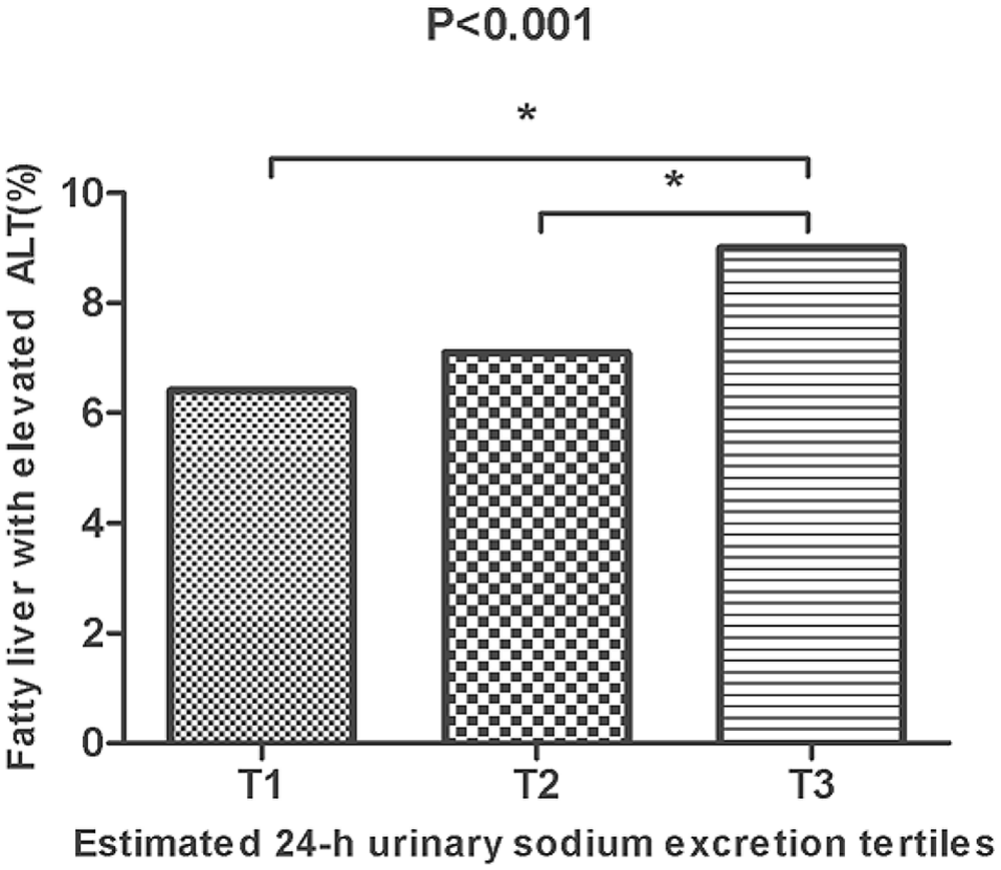
Association of estimated 24-h sodium excretion with prevalence of NAFLD assessed by FLI and elevated serum ALT levels. Elevated ALT levels were defined as >33 IU/L for males and >25 IU/L for females. * P-value < 0.05.

**Fig 2:**
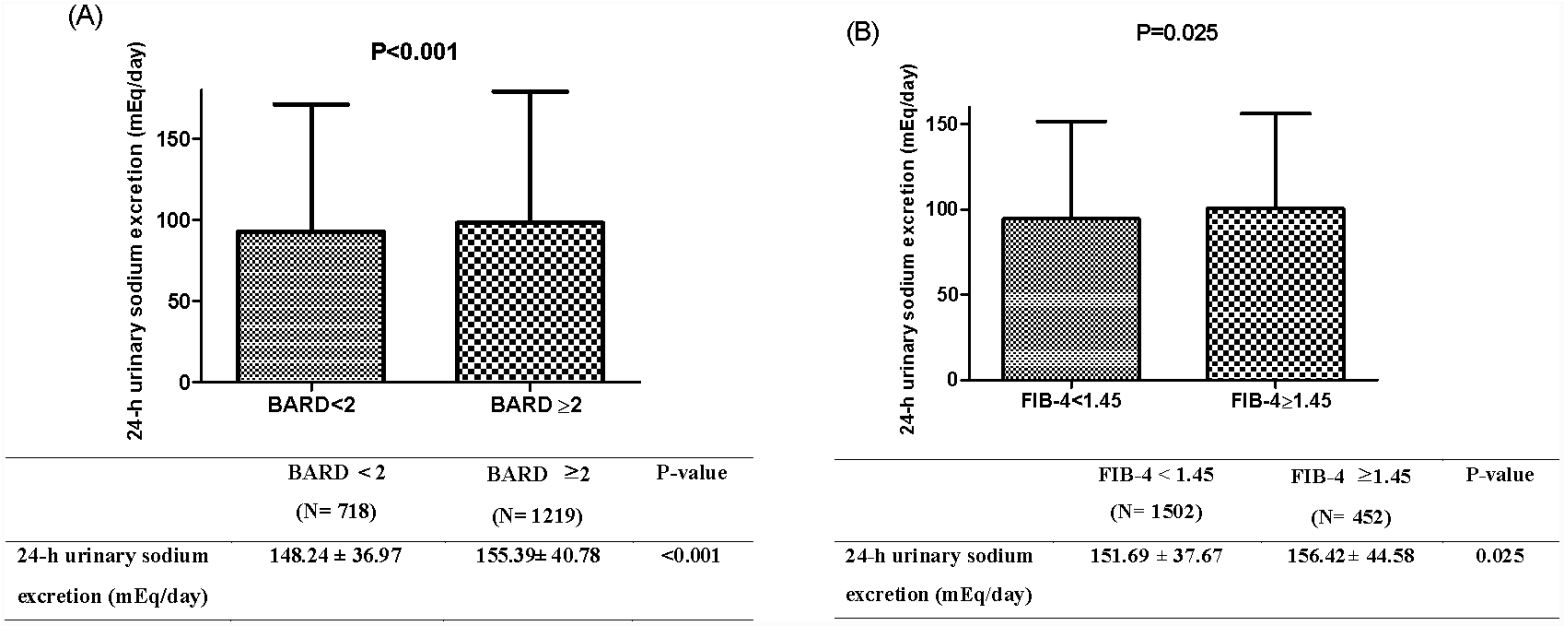
Differences in estimated 24-h urinary sodium excretion levels among groups with and without hepatic fibrosis defined by BARD score (A) and FIB-4 (B) in subjects with NAFLD. ** Data presented as mean + standard deviation.

Since ALT elevation corresponds to the severity of NAFLD, we also investigated NAFLD assessed by FLI (≥ 60) with elevated ALT levels (> 33 IU/L for males and > 25 IU/L for females) [20] as a surrogate marker for NASH. The prevalence of fatty liver with elevated ALT level increased in a graded manner over the estimated 24-h urinary sodium excretion tertiles (Fig 1). To further evaluate the relationship between estimated 24-h urinary sodium excretion and liver fibrosis, the noninvasive index of hepatic fibrosis, BARD score and FIB-4, were calculated in individuals with NAFLD assessed by FLI (≥ 60). When the validated cut-off points (BARD score ≥ 2 [21] and FIB-4 ≥ 1.45 [19]) to predict hepatic fibrosis were applied in this population, subjects with hepatic fibrosis were more likely to have higher estimated 24-h urinary sodium excretion than the other group (Fig 2A and 2B).

## Discussion

In this large cohort study, we found that high sodium intake, as assessed by estimated 24-hr urinary sodium excretion values, was independently associated with NAFLD in healthy, community-dwelling Koreans. Furthermore, we found that estimated 24-h urinary sodium excretion values were also associated with the severity of fatty liver, as determined by elevated markers of liver fibrosis. To the best of our knowledge, this is the first population-based study of the relationship between estimated 24-h urinary sodium excretion values and NAFLD.

Recently, some studies have shown that high salt intake is associated with metabolic syndrome and obesity. Yi et al. reported that high sodium intake was associated with higher odds of being obese, higher BMI, and greater body weight [22]. In addition, Baudrand et al. found that high sodium intake was also associated with insulin resistance and metabolic syndrome in a study population composed of 370 adults aged 18–85 years old [23]. Considering the known strong relationships between NAFLD, obesity and metabolic syndrome, we hypothesized that high salt intake, which can be assessed with estimated 24-h urinary sodium excretion values, might be also associated with NAFLD. There has been no study demonstrating the relationship between high sodium intake and NAFLD, and so we investigated this hypothesis in community-dwelling Korean adults.

In our study, we observed a significant association between high salt intake, expressed as estimated 24-h urinary sodium excretion values, and NAFLD. This association remained significant even after adjusting for body fat and insulin resistance reflected by HOMA-IR. From this finding, we speculate that high salt intake was independently associated with NAFLD regardless of body fat and insulin resistance. The significant relationship between high salt intake and NAFLD could be explained by several possible mechanisms. First, high salt intake may result in the dysregulation of the renin—angiotensin system [24], leading consequently to the development of NAFLD and progression to NASH. Recently, dysregulation of the renin-angiotensin system has been suggested to play a key role in hepatic inflammation and fibrosis [25]. Activation of mineralocorticoid receptors induces free radical production and oxidative stress by increasing nicotinamide dinucleotide phosphate (NADPH) oxidase [26] and reducing the expression of glucose-6-phosphate dehydrogenase (G6PD), which is a key anti-oxidant [27]. Actually, inhibition of the renin-angiotensin system in experimental animal models downregulates pro-inflammatory/pro-fibrotic cytokines, reduces activation of hepatic stellate cells, attenuates oxidative stress and inhibits hepatic inflammation and fibrosis [28, 29]. Second, high salt intake is generally the result of consumption of high energy foods with a high salt content, such as cheese and chips, which consequently increase the total energy intake and ultimately increase body fat [7]. In addition, the production of inflammatory cytokines and oxidative stress, which are both induced by increased body fat, may also be involved in the development of NAFLD [30]. Likewise, our study demonstrated that as estimated 24-h urinary sodium excretion increased, obesity indices such as waist circumference, BMI and body weight also increased. However, in our study, the association between estimated 24-h urinary sodium excretion and NAFLD remained even after adjustment for daily total energy intake or fat intake and body fat. This finding suggests that high sodium itself might be associated with NAFLD independent of obesity.

Although a liver biopsy is the gold standard for the diagnosis of NAFLD and its severity, imaging modalities such as ultrasonography or computed tomography are more widely used in clinical practice due to the invasive nature of biopsies. Such radiological modalities can be inaccurate, however, because they involve subjective, operator-dependent examination and do not detect mild degrees of hepatic steatosis (< 25–30%) well [31]. Due to the lack of imaging data for NAFLD in our study, we adopted several well-validated scoring systems to detect NAFLD in the general population or advanced fibrosis in patients with NAFLD. Among the models for predicting NAFLD, HSI and FLI were applied because of their considerable accuracy (areas under the curve 0.86 in HSI and 0.84 in FLI) to detect NAFLD in Korean subjects [32, 33]. In addition, because advanced hepatic fibrosis is known to be hardly detected by imaging modalities without biopsy, we also used BARD score and FIB-4, which have been validated in patients with biopsy-proven NAFLD [18], as a surrogate marker of hepatic fibrosis. As a result, participants with higher sodium intake had a higher level of liver fibrosis markers reflected by BARD score and FIB-4. These results suggest that high salt intake might influence NAFLD and hepatic fibrosis. In addition, we found out that subjects with higher sodium intake also had higher prevalence of NAFLD with elevated ALT. From these findings, we speculate that high sodium intake is associated with higher inflammatory status of NAFLD.

Our study has several limitations. First, since the present study was cross-sectional rather than longitudinal, a causal relationship between high salt intake and NAFLD could not be definitively established. Second, because this study did not include hepatic imaging or biopsies, we used indirect methods to define NAFLD or advanced fibrosis based on several predictive models that have been well validated. However, other recent studies also showed some important findings using these non-invasive validated NAFLD/hepatic fibrosis predictive models to overcome the limitation of abdominal sonography based diagnosis [34]. Third, our results may not be generally applicable to non-Korean populations because the pattern of food intake may vary by ethnic group. Fourth, we could not collect complete information regarding the degree of physical activity and the kind of anti-hypertensive drug such as diuretics which could influence urinary sodium excretion. Finally, we could not have improved the accuracy of individuals’ sodium intake by directly calculating estimated 24-h urinary sodium excretion using spot urine from participants. Despite these limitations, however, our study also had noteworthy strengths. First, we analyzed data were collected from a large, nationwide survey of Koreans which might increase the reliability of results. Second, this is the first observational study that extensively investigated the association between high sodium intake and NAFLD severity as well as NAFLD. In addition, for the first time, our study showed a strong relationship between high sodium intake and NAFLD independently of individual’s other dietary pattern except sodium intake and body fat.

In conclusion, our study is the largest and the first population-based study to examine associations between high salt intake and NAFLD in Korean adults. High salt intake assessed by increased estimated 24-h urinary sodium excretion was closely associated with elevated risks of NAFLD and NASH in healthy Koreans. In addition, the relationship between NAFLD and high dietary sodium intake was significant, independent of body fat and insulin resistance. Further large-scale prospective studies are needed to confirm the possible effect of high salt intake on NAFLD and identify the biological mechanisms underlying this association.

## Supporting Information

S1 Dataset Dataset. (ZIP)
